# TypA Interacts with ExsA to Suppress Type III Secretion System in *Pseudomonas aeruginosa*

**DOI:** 10.3390/microorganisms14091942

**Published:** 2026-09-02

**Authors:** Liwen Yin, Yiming Li, Xuetao Gong, Peishan Chen, Weihui Wu, Un-Hwan Ha, Shouguang Jin, Yongxin Jin

**Affiliations:** 1 State Key Laboratory of Medicinal Chemical Biology, Key Laboratory of Molecular Microbiology and Technology of the Ministry of Education, Department of Microbiology, College of Life Sciences, Nankai University, Tianjin 300071, China; yinliwen2022@163.com (L.Y.); mitchell.li@foxmail.com (Y.L.); 1120220627@mail.nankai.edu.cn (X.G.); wuweihui@nankai.edu.cn (W.W.); nksjin@nankai.edu.cn (S.J.); 2 Tianjin Key Laboratory of Acute Abdomen Disease Associated Organ Injury and ITCWM Repair, Institute of Acute Abdominal Diseases, Tianjin Nankai Hospital, Tianjin 300100, China; 3 Department of Biotechnology and Bioinformatics, Korea University, Sejong 30019, Republic of Korea; haunhwan@korea.ac.kr

**Keywords:** *P. aeruginosa*, T3SS, TypA, ExsA

## Abstract

*Pseudomonas aeruginosa* is a nosocomial pathogenic bacterium that causes a wide range of human infectious diseases. The type III secretion system (T3SS) serves as a key virulence determinant underlying the pathogenesis of this bacterium in acute infections. As the master transcriptional activator of T3SS, ExsA binds to target promoter regions and modulates the expression of all currently identified T3SS genes. In this study, we identified tyrosine phosphoprotein A (TypA) as a repressor that restricts expression of the T3SS in *P. aeruginosa*. TypA interacts with ExsA to block its binding to target promoters, thereby inhibiting T3SS expression. We show that the *typA* expression is induced in response to low calcium, low temperature, a biofilm lifestyle, and direct contact with host cells. Additionally, the absence of TypA caused a growth defect in *P. aeruginosa* at low temperatures. Collectively, these data confirm the significant role of TypA and reveal a novel molecular mechanism by which *P. aeruginosa* regulates T3SS.

## 1. Introduction

As a common nosocomial pathogen, *P. aeruginosa* causes various human infectious diseases, particularly in people with cystic fibrosis, burn injuries, and immunodeficiency [1]. The virulence determinants of *P. aeruginosa* are multifactorial, including biofilm formation, secreted toxins, and various forms of motility effects [2]. Among them, the type III secretion system (T3SS) is a key virulence factor contributing to bacterial pathogenesis in acute infections [2]. *P. aeruginosa* exploits the T3SS machinery to translocate four well-known effectors, ExoT, ExoS, ExoY and ExoU, directly from the bacterial cytosol into eukaryotic target cells, preventing phagocytic clearance by host immune cells [3].

Expression of the T3SS in *P. aeruginosa* is tightly regulated and stimulated by various environmental signals, including host cell contact, calcium limitation and serum exposure [4,5]. Without stimulatory signals for T3SS, ExsE interacts with ExsC, and ExsA remains trapped via binding by ExsD. In response to inducing cues, ExsE is secreted/translocated through T3SS, resulting in ExsC preferentially binding ExsD and liberating ExsA to turn on T3SS [6]. The entire T3SS gene set is activated by ExsA, which is a T3SS master regulator belonging to the AraC/XylS protein family [6]. ExsA binds to its consensus recognition sequence on DNA and recruits RNA polymerase to the promoters to activate the T3SS gene expression [7]. Many upstream regulators control *exsA* expression directly or indirectly at the transcriptional and/or translational levels [6]. Additionally, some proteins interfere with ExsA’s regulatory activity to regulate T3SS expression. ExsD, an anti-activator protein, binds directly to ExsA in a 1:1 complex, inhibiting ExsA homodimer assembly and impairing its capacity for promoter binding [8,9,10,11]. PtrA, a copper-inducible protein, directly interacts with ExsA to suppress T3SS expression [12]. However, the existence of other proteins that interact with and inhibit ExsA function remains unknown.

TypA (tyrosine phosphoprotein A), also known as BipA (BPI-inducible protein A), was initially characterized as a protein responsive to bactericidal/permeability-increasing protein (BPI), a neutrophil-secreted protein from humans that functions as a cationic antimicrobial [13,14]. TypA participates in the regulation of virulence factors and stress responses in various bacteria [15,16,17,18]. Reports indicate that TypA undergoes tyrosine phosphorylation in specific bacterial strains [14].

In this study, we identified TypA as a T3SS repressor in *P. aeruginosa*. TypA interacts with ExsA, interfering with ExsA’s binding to its target promoters, thereby inhibiting T3SS expression. The expression of *typA* is induced in response to low calcium, low temperature, during biofilm formation, and upon direct contact with host cells. In addition, we also investigated the role of TypA in *P. aeruginosa* growth at low temperature.

## 2. Materials and Methods

### 2.1. Microbial Strains, Plasmids and Antibiotics

*P. aeruginosa*, *Escherichia coli* strains, and plasmids used in the present study are listed in Table S2. Primers for PCR are listed in Table S3. Bacteria were routinely grown in Luria-Bertani (LB) medium (10 g/L tryptone, 5 g/L yeast extract, and 5 g/L NaCl) at 37 °C with constant shaking at 200 revolutions per minute or on LB agar plates with agar concentration 15 g/L. Antibiotics were supplemented to medium at the concentrations listed below when needed: tetracycline (Tc) 50 μg/mL, gentamicin (Gm) 50 μg/mL, carbenicillin (Cb) 150 μg/mL for *P. aeruginosa*; tetracycline (Tc) 10 μg/mL, kanamycin (Kan) 25 µg/mL, gentamicin (Gm) 10 μg/mL, ampicillin (Amp) 100 μg/mL and chloramphenicol (Chl) 30 μg/mL for *E. coli*.

### 2.2. Genetic Techniques

For deletion of the *typA* gene, the upstream and downstream flanking fragments of *typA* were PCR-amplified with specific primers (Table S3) to generate the knockout plasmid pEX18Tc-*typA*. Briefly, the PCR-derived DNA was digested with the appropriate restriction enzymes, followed by directional ligation into pEX18Tc. Then, pEX18Tc-*typA* was transferred to *P. aeruginosa* strains through conjugal transfer using *E. coli* S17-1 as the donor strain. *typA* gene deletion was performed via homologous recombination. Briefly, single crossover mutants were initially screened, and subsequent double crossover strains were further obtained using established procedures [19]. The target *typA* knockout mutant was validated using PCR amplification and sequencing analysis.

Plasmid pUCP21-*exsA*-Flag-His was constructed via PCR amplification of the *exsA* gene utilizing primers *exsA*-Flag-His-F/R (Table S3) with genomic DNA from the PAK strain serving as template. Purified PCR amplicons were cleaved with *Sac*I-*Eco*RI and then ligated into pUCP21. Plasmids pUCP24-*typA*-Flag, pUC18T-mini-Tn7T-*typA*-Flag, pMMB67EH-*exsA*-His, pBT-*exsA*, pTRG-*typA* and pTRG-*exsD* were generated using similar strategies.

Plasmid pET28a-*typA*-Flag-His was generated via PCR amplification of the *typA*-Flag fragment from the plasmid pUCP24-*typA*-Flag using primers *typA*-Flag-His-F and *typA*-Flag-His-R (Table S3). Purified PCR products were cloned into the vector pET28a using the One Step Cloning Kit (Vazyme, Nanjing, China). Similarly, the PCR product amplified using primers GST-A-F and GST-A-R (Table S3) from pMMB67EH-GST-*exsA*-His was inserted into pET28a to obtain plasmid pET28a-GST-*exsA*-His by the One Step Cloning Kit (Vazyme). Plasmid pMMB67EH-GST-*exsA*-His was constructed via replacement of *cspc* with *exsA* in plasmid pMMB67EH-*cspC*-GST [20].

### 2.3. Co-Immunoprecipitation Assay

To obtain ExsA interacting proteins, a Co-Immunoprecipitation assay was performed as described previously [21]. Briefly, the PAKΔ*exsA* strain containing pUCP21 (EV) or pUCP21-*exsA*-Flag-His was grown in 300 mL LB medium plus carbenicillin (150 μg/mL) to an OD_600_ of about 1.0 at 37 °C; then, bacterial pellets were obtained through centrifugation prior to resuspension in lysis buffer (46.6 mM Na_2_HPO_4_, 3.4 mM NaH_2_PO_4_, 0.3 M NaCl, pH 8.0). Subsequent procedures were maintained on ice or at 4 °C. The cell samples were sonicated and centrifuged to eliminate cellular debris. The supernatants (soluble fraction) were then incubated with nickel–nitrilotriacetic acid–agarose beads (Ni-NTA, Qiagen, Hilden, Germany) according to the manufacturer’s recommendation. After washing three times with lysis buffer containing 20 mM imidazole, the bead-retained proteins were sequentially eluted using lysis buffer containing 100, 200, 300 mM imidazole. After SDS-PAGE separation, protein bands were stained with Coomassie blue for visualization. Protein bands retained by beads in sample PAKΔ*exsA*/pUCP21-*exsA*-Flag-His, but not in PAKΔ*exsA*/pUCP21, were excised and analyzed by mass spectrometry.

To determine the interaction between TypA and ExsA, the Co-Immunoprecipitation assay (Co-IP) was carried out as previously documented with marginal modifications [22]. The His-tagged ExsA or GFP protein on the plasmid and Flag-tagged TypA protein integrated into the chromosome were co-expressed in PAKΔ*exsA*. At initial growth to an OD_600_ of approximately 0.6, IPTG was added to a final concentration of 1 mM to induce the production of proteins at 37 °C for 3 h. Harvested bacterial pellets were resuspended in ice-cold lysis buffer, subjected to sonication and centrifuged at 12,000× *g*, 4 °C for 10 min to discard insoluble cellular debris. Then, 20 μL of the supernatant was saved to generate the input Western blot sample. The Ni-NTA agarose beads, which had been pre-equilibrated using lysis buffer, were added to the remaining supernatant. After 1 h of incubation at 4 °C, the beads underwent three washes using lysis buffer supplemented with 20 mM imidazole. Proteins retained on the beads were then examined by Western blot analysis after SDS-PAGE.

### 2.4. Western Blot Assay

Indicated samples from equal amounts of bacterial samples were supplemented with loading buffer, heated at 99 °C for 10 min, and subsequently resolved on a 12% SDS-PAGE. Proteins were transferred to PVDF membrane, immunoblotted with primary antibodies targeting ExoS, FLAG (Sigma, Livonia, MI, USA), His (Millipore, Burlington, MA, USA), GST (Applygen, Beijing, China) or RpoA (RNAP, Abcam, Cambridge, MA, USA), and then with the corresponding secondary antibody Anti-Rabbit IgG and Anti-Mouse IgG purchased from Promega. Target protein signals were detected using a Millipore ECL Plus detection kit.

### 2.5. Cytotoxicity Assay

Cell cytotoxicity assays were conducted in accordance with a previous description [23]. The A549 cells were plated at 2 × 10^5^ cells into individual wells of 24-well plates the night before infection at 37 °C under 5% CO_2_ conditions. PBS-resuspended log-phase bacteria were used to challenge A549 cells at a multiplicity of infection (MOI) of 50. The cytotoxicity was detected via crystal violet staining after two hours infection. Specifically, the medium was discarded, and each well underwent two washes with PBS. Crystal violet staining solution (0.25%) was added to each well to fully cover the cells, and incubation proceeded at room temperature for 15 min. After two washes with distilled water, 200 µL of destaining buffer containing 10% acetic acid and 40% methanol was added to each well. The dissolved eluates were quantified at 590 nm with a Varioskan Flash microplate reader (Thermo Scientific, Waltham, MA, USA).

### 2.6. Quantitative Real-Time PCR Assay

Total RNA from the indicated *P. aeruginosa* strains was extracted with a bacterial total RNA kit (Zomanbio, Beijing, China). Potential DNA was further removed using DNase I (Takara, Dalian, China). A total of 1 μg RNA was used for the subsequent synthesis of cDNA using reverse transcriptase mix (Vazyme, Nanjing, China). The resulting cDNA was analyzed via quantitative real-time PCR with SYBR qPCR Mix (Vazyme, Nanjing, China). Relative mRNA levels were quantified using the 2^−ΔΔCt^ method. The *rpsL* gene, which encodes a ribosomal 30S subunit protein, was utilized as an internal control for normalization.

To examine transcriptional levels responding to low temperature, bacteria were propagated at 37 °C up to an OD_600_ of 1.0 before temperature downshift to 20 °C with 30 min prolonged incubation. For host contact, bacteria at log growth phase were centrifuged, normalized by OD_600_ in PBS and incubated with or without A549 cells for 1 h. For the biofilm lifestyle, the biofilm was formed in a 6-well plate and collected with a scrape. Planktonic PAK cells were harvested from shake-tube cultures. Total RNA was isolated, and real-time qPCR was performed as described above.

### 2.7. Bacterial Two-Hybrid Assay

Assays for interprotein interaction using the bacterial two-hybrid system were conducted following the manufacturer’s provided guidelines (Stratagene, La Jolla, CA, USA). Recombinant pBT and pTRG derivative plasmids were simultaneously electroporated into the *E. coli* RS strain. Plasmid-bearing strains were cultured in LB medium containing chloramphenicol and tetracycline at 30 °C overnight, then diluted into LB medium with antibiotics. At an OD_600_ of 0.6, IPTG was supplemented at a final concentration of 0.1 mM to induce fusion protein production over a 4 h incubation. Interactions were quantified by β-galactosidase assays.

### 2.8. Protein Purification

To purify the recombinant proteins, the *E. coli* strain BL21 (DE3) containing pET28a-*exsA*-His or pET28a-*typA*-Flag-His was cultured at 37 °C. At an OD_600_ of 0.6, IPTG (0.1 mM) was supplemented, and incubation continued at 16 °C overnight. After centrifugation at 8000× *g* for 10 min, the bacterial pellets were resuspended in lysis buffer containing 50 mM Na_2_PO_4_ and 0.3 M NaCl. The suspension was sonicated, and insoluble material was discarded by centrifugation. Clarified lysates containing soluble proteins were incubated with a Ni-NTA column (Qiagen, Hilden, Germany). After three rounds of washing using lysis buffer containing 50 mM imidazole, the bead-retained proteins were eluted using 300 mM imidazole in lysis buffer. Proteins obtained through the above procedures were tested by SDS-PAGE. GST-His and GST-ExsA-His were purified using a similar strategy.

### 2.9. Electrophoretic Mobility Shift Assay (EMSA)

EMSA was carried out according to prior methods with minor alterations [24]. The *exsC* promoter and *algD* gene fragments were PCR-amplified using specific primers (Table S3). A total of 40 ng DNA probe was mixed with purified recombinant ExsA-His and/or TypA-Flag-His or GST-His proteins in a 20 μL reaction system (10 mM Tris-HCl, 5 mM MgCl_2_, 5 mM KCl, 1 mM dithiothreitol, pH 7.5). The binding reaction was kept at 4 °C for 25 min, followed by a 5 min incubation at 37 °C to promote protein–DNA complex formation. After mixing with loading buffer (5% glycerol, 0.01% bromophenol blue), the samples were separated via electrophoresis on an 8% native polyacrylamide gel prepared in 1 × TBE buffer. The gel was pre-run for 1 h to establish a stable electric field. The 1 × TBE running buffer contained 89 mM Tris, 89 mM boric acid and 2 mM EDTA (pH 8.3). Electrophoresis was conducted at 10 mA for about 1.5 h on ice, then the gel was stained by soaking in 0.5 µg/mL EB staining solution for 10 min at ambient temperature. Finally, the bands were visualized using ChemiDoc^TM^ XRS+ (Bio-Rad, Hercules, CA, USA).

### 2.10. Glutathione-S-Transferase Pull-Down (GST Pull-Down) Assay

One nmol of purified GST-His or GST-ExsA-His protein was incubated with 50 μL GST-tag Purification Resin (Beyotime, Shanghai, China) at 4 °C for 1 h. The resin was subsequently washed five times using 1 mL lysis buffer per wash and then incubated with 1 nmol TypA-Flag-His at 4 °C for 1 h. After centrifugation at 1000× *g* for 30 s, the collected resin complex was washed five times using 1 mL lysis buffer. The resin was collected and solubilized in SDS loading buffer. Western blot analysis was used to examine the collected proteins.

### 2.11. Mouse Acute Pneumonia Model

Mouse acute pneumonia modeling was established as described in earlier research [23]. To examine bacterial pathogenicity, *P. aeruginosa* at an OD_600_ of approximately 1.0 was harvested and washed twice using PBS. Female BALB/c mice aged 6–8 weeks old (Vital River, Beijing, China) were anesthetized using 100 μL 7.5% chloral hydrate via intraperitoneal injection. Ten microliters of bacterial suspension were intranasally inoculated into each nostril, resulting in a total inoculum of 1 × 10^7^ CFU per mouse. After 12 h of infection, all mice were euthanized via CO_2_ inhalation. The lung tissues were harvested and homogenized in 1% proteose peptone. Finally, the bacterial loads were quantified by CFU plating assay.

## 3. Results

### 3.1. Identification of Candidate Proteins Interacting with the Master Activator ExsA

**Figure 1:**
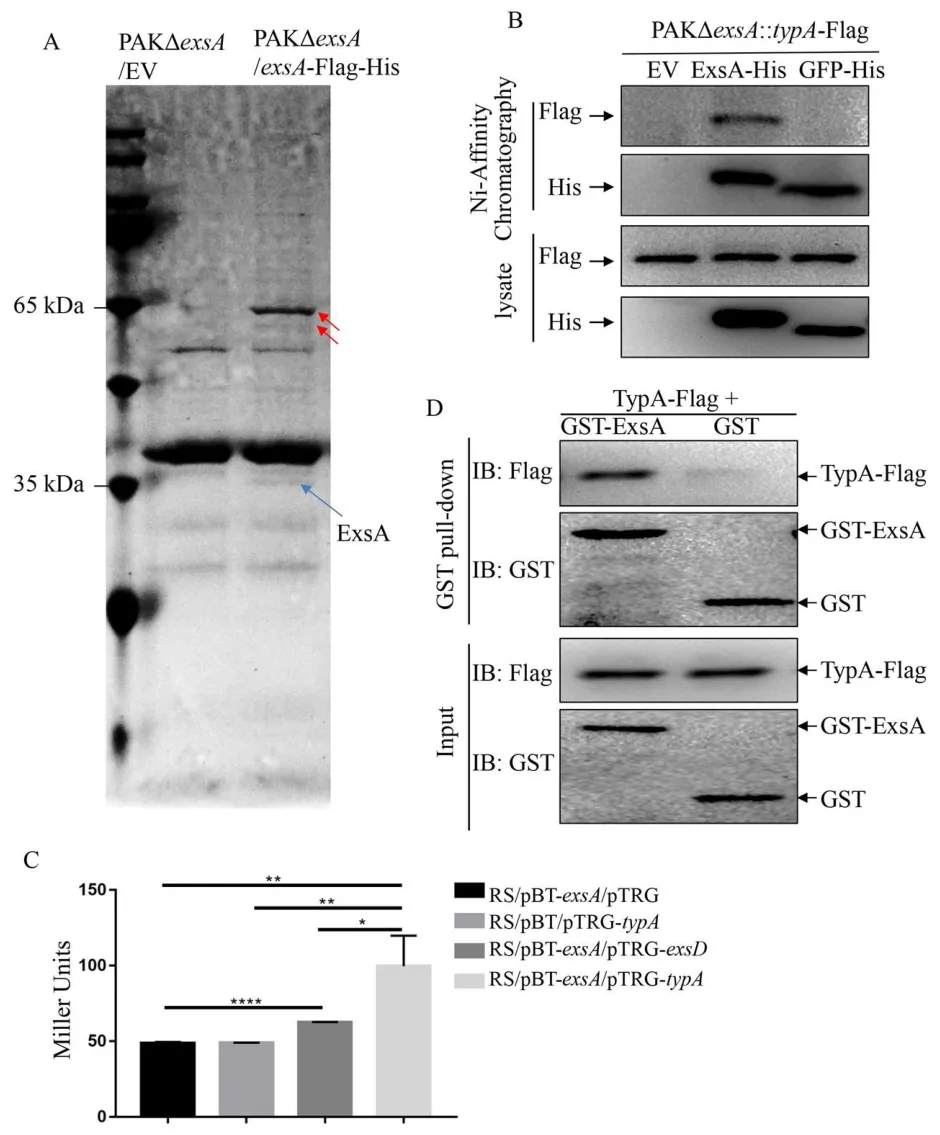
Identification of ExsA interacting proteins. (**A**) The PAKΔ*exsA* mutant containing the *exsA*-Flag-His fusion on pUCP21 or the empty vector (EV) was grown to an OD_600_ of 1.0 in LB. Proteins co-immunoprecipitated from bacterial lysates with Ni-NTA agarose beads were eluted with 300 mM imidazole. ExsA-Flag-His and associated proteins were separated on SDS-PAGE and stained with Coomassie blue. Protein bands different from the control sample (PAKΔ*exsA*/EV) were indicated with red arrows, excised, pooled into one group, and analyzed by LC-MS/MS. (**B**) The PAKΔ*exsA* mutant carrying chromosomally integrated *typA*-Flag and plasmid-borne *exsA*-His, GFP-His or empty vector was grown to an OD_600_ of 0.6 and treated with 1 mM IPTG for 3 h. Bacteria were sonicated and subjected to chromatography with Ni-NTA agarose. His-tagged ExsA/GFP and Flag-tagged TypA were detected with Western blot using His and Flag antibodies. (**C**) Examination of protein–protein interactions between TypA and ExsA using the BacterioMatch Two-hybrid System with ExsA–ExsD interaction as a positive control. *, *p* < 0.05; **, *p* < 0.01; ****, *p* < 0.0001, by Student’s *t*-test. (**D**) Testing the interaction between TypA and ExsA using a GST pull-down assay. GST-binding beads coated with GST-ExsA-His or GST-His were incubated with TypA-Flag-His. After washing with lysis buffer, proteins were examined by Western blot with antibodies against GST or Flag.

ExsA functions as the master regulator that activates the transcription of whole T3SS genes in *P. aeruginosa*, and many upstream regulatory factors govern T3SS expression via direct and indirect modulation of ExsA [6]. To investigate candidate proteins interacting with the ExsA, we expressed a functional ExsA-Flag-His fusion protein in the PAKΔ*exsA* strain under T3SS non-inducing condition and conducted Co-immunoprecipitation (Co-IP) to uncover ExsA interacting partners. The co-purified protein samples were fractionated by SDS-PAGE and visualized via Coomassie brilliant blue staining. As displayed in Figure 1A, two distinct protein bands were observed in the PAKΔ*exsA*/ExsA-Flag-His strain, but not in the vector control strain PAKΔ*exsA*/EV. Mass spectrometric (MS) analysis of the two bands recovered from the SDS-PAGE gel revealed a mixture of 417 proteins (Table S1). Consistent with previous reports [9,11,25], ExsD was detected in the MS analysis. Notably, TypA, a 66.9 kD protein, was also identified in the MS analysis. TypA, also known as BipA, has been reported to participate in modulating the virulence of *P. aeruginosa* PA14 [26].

To validate the potential interaction between ExsA and TypA, we overexpressed functional ExsA-His fusion protein in the PAKΔ*exsA* strain carrying a chromosomally integrated C-terminal Flag-tagged TypA. Recombinant His-tagged ExsA protein was purified by Ni affinity purification. As displayed in Figure 1B, TypA-Flag was successfully co-purified with ExsA-His, whereas no binding was detected for the empty vector (EV) or the GFP-His control group, confirming the interaction of ExsA with TypA.

A BacterioMatch Two-hybrid System was further used to study the direct interaction between TypA and ExsA. The *typA* gene was inserted into a target vector, while the *exsA* gene was cloned into the bait vector. As a positive control, the anti-ExsA protein ExsD coding gene was inserted into the target vector. The data in Figure 1C reveal that β-galactosidase activity was increased when TypA and ExsA were co-expression, even higher than that of the positive control (ExsA and ExsD), confirming direct TypA-ExsA physical interaction.

To further validate the direct interaction, a GST pull-down assay was performed by incubating GST-ExsA-His or GST-His with TypA-Flag-His (with molecular mass about 70 kD, Figure S1) purified from *E. coli*. The reaction mixtures were subsequently purified with GST affinity beads, and bead-bound proteins were examined by Western blot. As shown in Figure 1D, TypA-Flag-His was retained with GST-ExsA-His, but not with GST-His, which further validates the direct interaction between TypA and ExsA.

### 3.2. TypA Prevents ExsA Binding to the Promoter of P_exsC_

**Figure 2:**
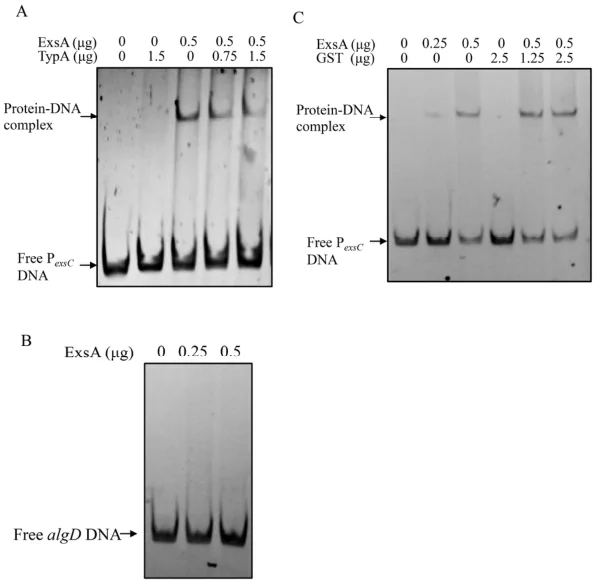
TypA prevented ExsA from binding to the *exsC* promoter as determined by EMSA. Increasing amounts of purified TypA-Flag-His (**A**) or GST (**C**) were incubated with 0.5 µg ExsA-His and 40 ng of DNA fragments corresponding to the *exsC* promoter. Incubation of ExsA-His with *algD* DNA fragments serves as a negative control for ExsA’s binding to P*_exsC_* (**B**). The mixtures were electrophoresed on an 8% native PAGE gel, and the bands were detected under UV light after ethidium bromide staining.

ExsA drives T3SS transcription by directly binding to the promoter sequences of T3SS operons [10,27]. The direct interaction of TypA with ExsA prompted us to test whether this interaction prevents ExsA from binding to the promoter regions of T3SS. Accordingly, electrophoresis mobility shift assays (EMSA) were conducted using the *exsC* promoter fragment and the purified recombinant ExsA-His protein. Consistent with previous studies [10,27], upon incubation with ExsA-His protein, a clear shifted band was detected for the *exsC* promoter fragment, but not for the negative control *algD* fragment (Figure 2A,B). Notably, the addition of TypA protein into the reaction system, but not GST, decreased the binding of ExsA-His to the P*_exsC_* promoter (Figure 2A,C), and the effect exhibited a dose-dependent manner (Figure 2A). These findings indicate that the direct binding of TypA prevents the ExsA from binding to the T3SS gene promoters.

### 3.3. Deficiency of typA Increases the T3SS Expression in P. aeruginosa

**Figure 3:**
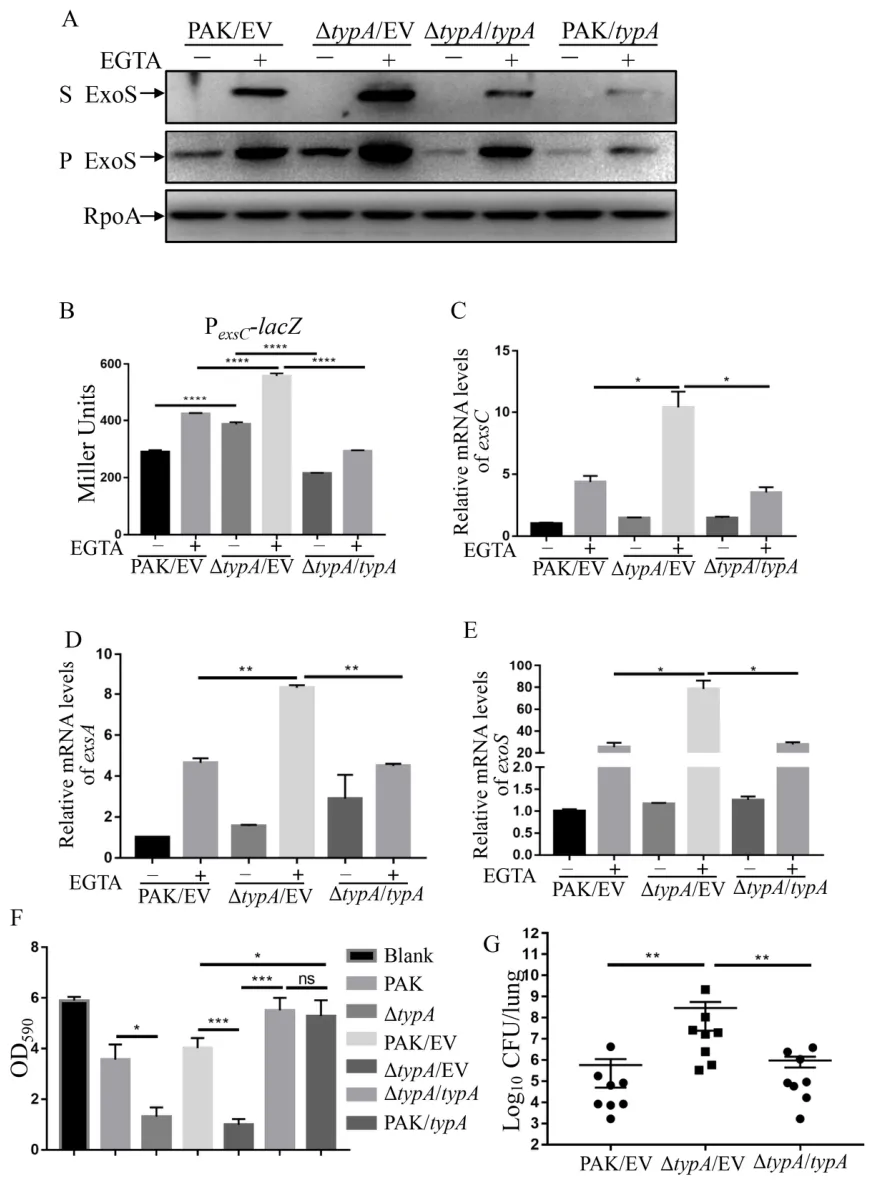
T3SS and cytotoxicity were upregulated in the PAKΔ*typA* mutant. (**A**) Expression and secretion of ExoS in PAK/pUCP24 (PAK/EV), Δ*typA*/pUCP24, Δ*typA*/pUCP24-*typA*-Flag (Δ*typA*/*typA*) and PAK/pUCP24-*typA*-Flag (PAK/*typA*). Bacterial cells were grown to an OD_600_ of 1.0 in LB medium with or without 5 mM EGTA. Protein samples in supernatants (S) and pellets (P) from equivalent bacterial cells were separated on 12% SDS-PAGE gels and probed with ExoS or RpoA antibodies. (**B**) Indicated bacterial strains containing the P*_exsC_*-*lacZ* transcriptional reporter plasmid were cultured to an OD_600_ of 1.0 in LB with or without 5 mM EGTA and subjected to β-galactosidase assays. Each assay was performed in triplicate, and error bars indicate standard deviations. ****, *p* < 0.0001 by Student’s *t*-test. (**C**–**E**) Relative mRNA levels of *exsC* (**C**), *exsA* (**D**) and *exoS* (**E**) in the indicated strains. Total RNA was isolated from bacterial cells at an OD_600_ of 1.0, and the relative mRNA levels of *exsC*, *exsA* and *exoS* were determined by real-time qPCR using the ribosomal encoding gene *rpsL* as an internal control. *, *p* < 0.05; **, *p* < 0.01 by Student’s *t*-test. (**F**) Cytotoxicity to A549 cells of indicated bacterial strains. A549 cells were incubated with the indicated strains at an MOI of 50. Two hours after infection, cells attached to the 24-well plate were washed with PBS and stained with crystal violet. The cell-associated crystal violet was dissolved in destaining buffer and quantified by reading the OD_590_ value. A549 cells with no bacterial incubation (Blank) served as a control. ns, not significant, *, *p* < 0.05; ***, *p* < 0.001, by Student’s *t*-test. (**G**) Mice were inoculated intranasally with 1 × 10^7^ CFU bacteria of the indicated strains. At 12 hpi, the mice were sacrificed and lungs were isolated and homogenized. The bacterial loads were determined by serial dilution and plating. The central bar indicates the mean, and the error bar indicates standard error. **, *p* < 0.01, by the Mann–Whitney test.

To further determine the contribution of *typA* to the expression of T3SS genes in *P. aeruginosa*, *typA* was deleted in the wild-type (WT) PAK strain. Western blot assay showed that the expression and secretion of ExoS increased in PAKΔ*typA* compared to the wild-type strain PAK under T3SS-inducing conditions with 5 mM EGTA as a calcium chelator (Figure 3A). Complementation with a *typA*-expressing plasmid in PAKΔ*typA* decreased the production and secretion of ExoS (Figure 3A). To further verify the upregulated T3SS in the Δ*typA* strain, promoter-*lacZ* fusion reporter assays and real-time qPCR assays were conducted to examine the transcriptional levels of the T3SS. As shown in Figure 3B, P*_exsC_*-*lacZ* transcriptional fusion reporter activity demonstrated a strong induction when both PAK and Δ*typA* strains were grown in medium containing 5 mM EGTA. Additionally, the Δ*typA* strain displayed significantly increased β-galactosidase activity relative to the PAK strain, regardless of T3SS induction conditions, which could be complemented by a *typA* clone in trans. Consistent with this result, real-time qPCR showed significantly elevated levels of the *exsC*, e*xsA* and *exoS* mRNA in Δ*typA* compared to the PAK or Δ*typA* complementation strain under the T3SS-inducing condition (Figure 3C–E).

Given that T3SS exerts a major effect on the cytotoxicity of *P. aeruginosa* [28], the functional association between TypA and T3SS led us to investigate the role of TypA in cytotoxicity. Accordingly, we infected A549 human lung adenocarcinoma epithelial cells [29] with PAK, Δ*typA*, and Δ*typA* complement strains and measured cytotoxicity using a crystal violet staining assay. As displayed in Figure 3F, loss of the *typA* gene in PAK resulted in higher cytotoxicity, while complementation with a *typA* gene clone reversed the cytotoxicity. These findings further demonstrated that TypA is a negative regulator of the T3SS in PAK. Since TypA functions as a negative regulator of the T3SS, we tested the effects of *typA* overexpression on the expression of T3SS and bacterial cytotoxicity. As shown in Figure 3A,F, the ExoS expression and cytotoxicity decreased when *typA* was overexpressed in the PAK strain.

Since the T3SS is critical for *P. aeruginosa* virulence in acute infections, we further evaluated bacterial virulence using a mouse acute pneumonia model. Mutation of *typA* increased bacterial loads in the lungs, and complementation with a wild-type *typA* gene restored the bacterial loading (Figure 3G). Combined, all these findings demonstrate that TypA acts as a negative regulator on T3SS genes in PAK.

To exclude strain specificity, we deleted *typA* genes in PA14 and PAO1 strains, the other two lab strains of *P. aeruginosa*, and examined its effect on T3SS. As shown in Figure S2, both expression and secretion of ExoS were slightly increased in PAO1Δ*typA* compared to its parental strain, PAO1. Similarly, expression of ExoU displayed a slight increase in the PA14Δ*typA* strain compared to its wild-type strain, PA14 (Figure S2A,B). Consistent with this, real-time qPCR showed increased mRNA levels of *exsC*, *exsA*, *exoS* and *exoU* in Δ*typA* mutants compared to their respective parental strains PAO1 or PA14 under the T3SS-inducing condition (Figure S2C,D). Similarly, the absence of *typA* led to increased cytotoxicity in both PAO1 and PA14 backgrounds (Figure S2E,F). In summary, TypA functions as a negative modulator for the T3SS in *P. aeruginosa*.

### 3.4. Expression Patterns of typA in P. aeruginosa

**Figure 4:**
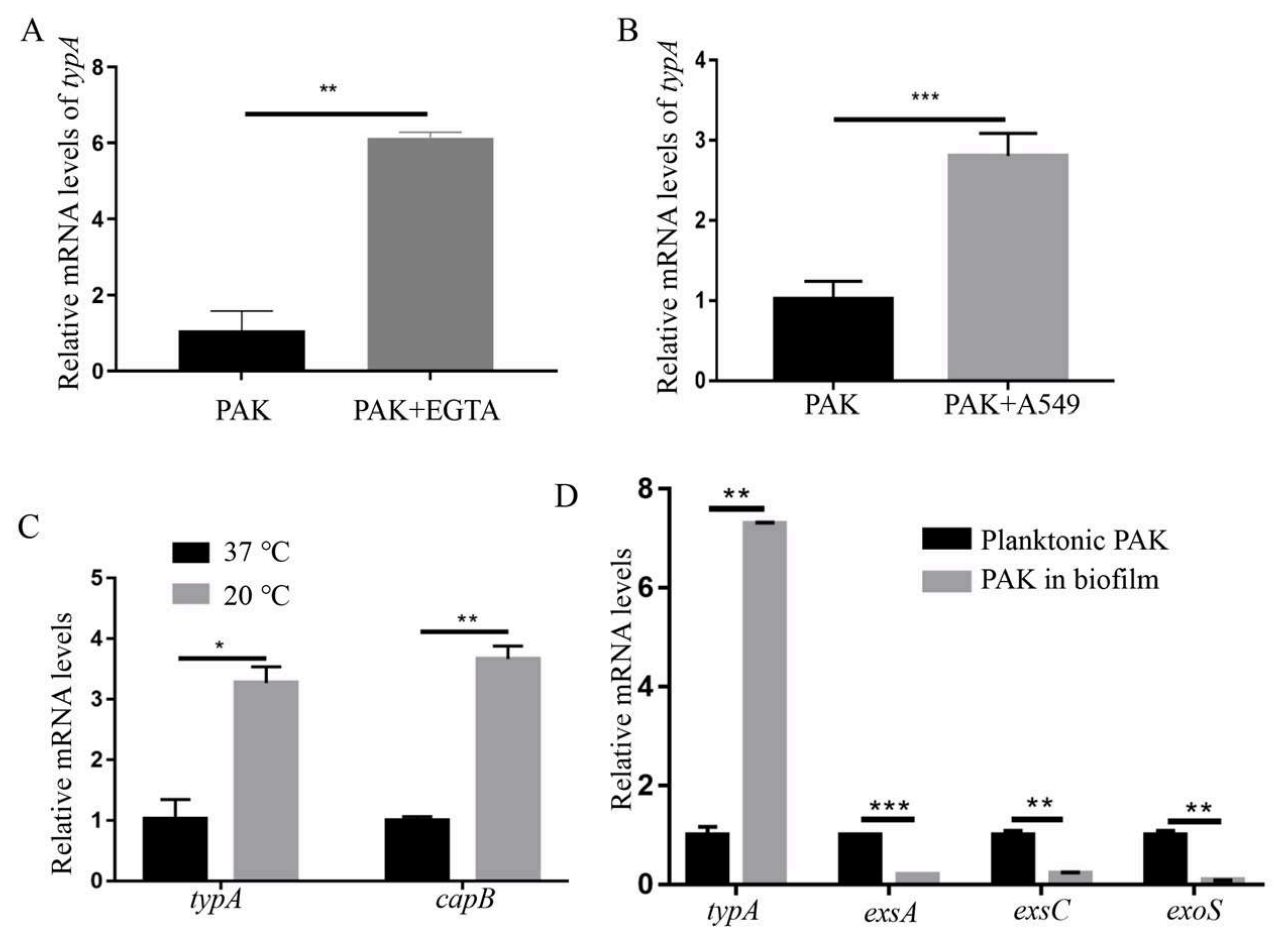
Expression patterns of *typA* in *P. aeruginosa*. Relative mRNA levels of *typA* in the PAK strain in response to EGTA addition (**A**), contact with A549 cells (**B**), low temperature (**C**) and biofilm growth style (**D**). Total RNA was isolated from bacterial cells at an OD_600_ of 1.0 (**A**–**C**) or growing in planktonic/biofilm lifestyle (**D**), and the relative mRNA levels of *typA*, *capB*, *exsA*, *exsC* and *exoS* were determined by real-time qPCR using the ribosomal encoding gene *rpsL* as an internal control. *, *p* < 0.05; **, *p* < 0.01; ***, *p* < 0.001 by Student’s *t*-test.

Since T3SS is stimulated by low calcium and host contact, and TypA serves as a suppressor of T3SS in *P. aeruginosa*, we sought to determine if the expression of *typA* is influenced by low calcium or host contact. Therefore, PAK was grown in LB medium with or without 5 mM EGTA or incubated with or without A549 cells, and RNAs were purified to determine the relative *typA* mRNA levels. As shown in Figure 4A,B, the mRNA levels of *typA* increased significantly when bacteria were grown under T3SS-inducing conditions, including induction with 5 mM EGTA and incubation with A549 cells.

Previous studies revealed an upregulation of T3SS genes in *P. aeruginosa* when the growth temperature was shifted to 37 °C from 28 °C or 25 °C [20,30]. Using real-time qPCR, we confirmed the decreased expression of T3SS genes when PAK was cultured at 20 °C compared to 37 °C (Figure S3). Given that *typA* expression is stimulated by low temperature in *E. coli* [31], and that TypA functions as a repressor for T3SS in *P. aeruginosa*, we further examined whether low temperature influences *typA* expression in *P. aeruginosa*. As a positive control, we examined the relative mRNA levels of a major cold-shock responsive gene *capB* (also known as *cspA*) using real-time qPCR, and it displayed a dramatic elevation once the temperature downshift (3.67-fold, Figure 4C). Similarly, the expression of *typA* was significantly induced at 20 °C compared to 37 °C (3.21-fold, Figure 4C). These results link increased TypA expression with decreased T3SS at low temperature, providing a potential explanation for the reduced T3SS at low temperature.

Biofilm formation is well known, and the expression of biofilm factors has an inverse correlation with T3SS in *P. aeruginosa* [32,33]. To test whether *typA* expression is influenced by the biofilm lifestyle, we collected bacterial cells from biofilm and planktonic cultures, purified RNAs, and determined the relative mRNA abundances of *typA* using real-time qPCR. As indicated in Figure 4D, *typA* was significantly upregulated in the biofilm lifestyle compared to the planktonic lifestyle, while T3SS genes *exsA*, *exsC* and *exoS* were significantly downregulated in the biofilm lifestyle.

### 3.5. Mutation of typA Impairs the Growth at Low Temperature in P. aeruginosa

**Figure 5:**
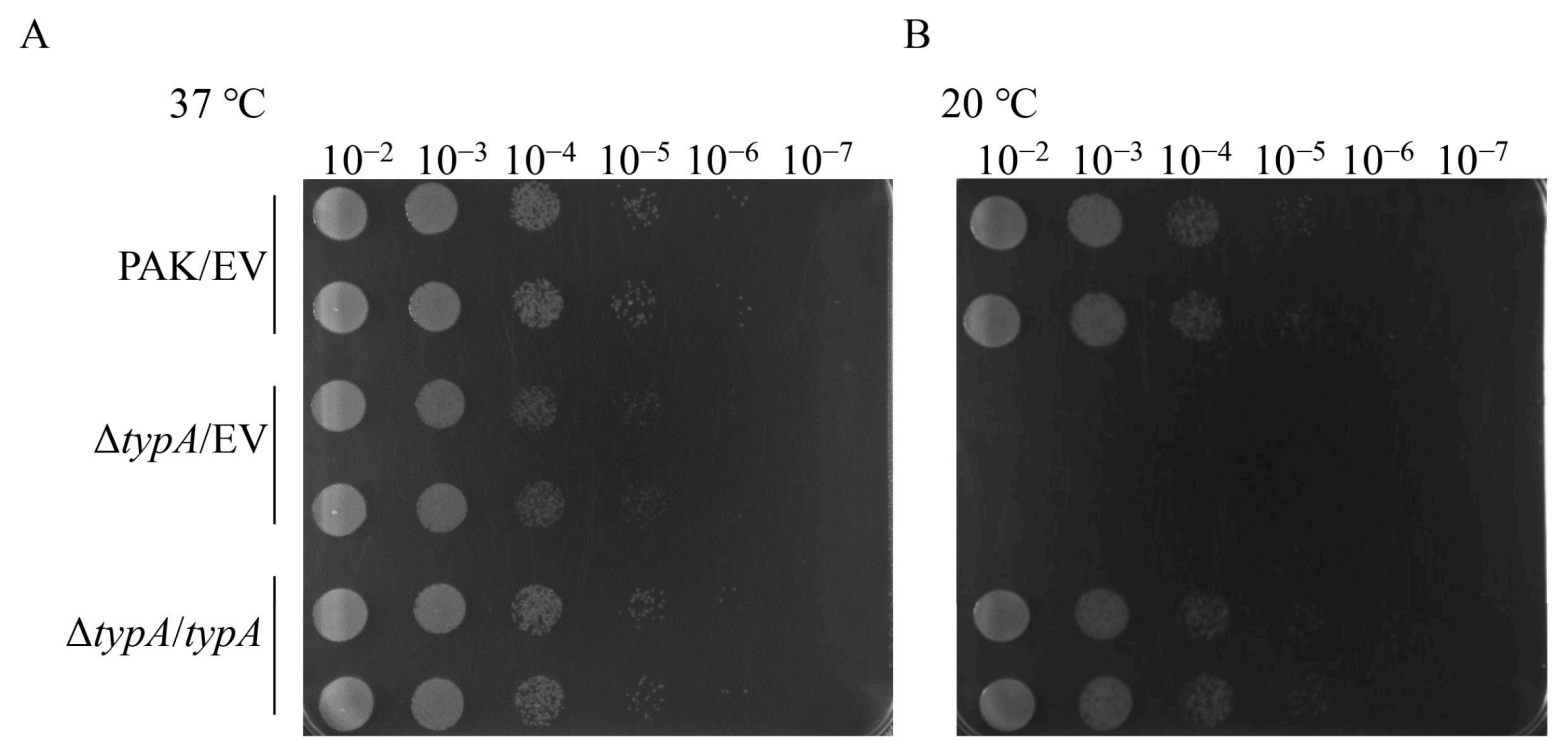
Role of *typA* in *P. aeruginosa* PAK cold adaptation. (**A**) 37 °C and (**B**) 20 °C growth assays of wild-type PAK/pUCP24 (PAK/EV), PAKΔ*typA*/pUCP24 (Δ*typA*/EV) and PAKΔ*typA*/pUCP24-*typA*-Flag (Δ*typA*/*typA*).

Given TypA has been reported to be essential for the cold adaption of *E. coli* K-12 [34], we investigated whether TypA affects the growth of *P. aeruginosa* PAK at 20 °C. Ten μL of 10-fold serially diluted bacterial culture was dropped onto LB agar medium and cultured separately at 37 °C or 20 °C. As observed in Figure 5, the *typA* mutant grew similarly with PAK on LB agar plates when incubated at 37 °C (Figure 5A) but displayed impaired growth when incubated at 20 °C (Figure 5B).

## 4. Discussion

In *P. aeruginosa*, transcription of the entire T3SS genes is highly dependent on the master regulator ExsA. Therefore, ExsA and its regulatory biological processes represent a key biomedical research focus. Previous studies have demonstrated that ExsD and PtrA can bind to ExsA and block ExsA-dependent T3SS expression [11,12]. In this study, we found that TypA interacts with ExsA to suppress the expression of T3SS genes in *P. aeruginosa*. We showed that the transcription of *typA* was elevated at 20 °C compared to 37 °C. We previously reported that the acetylation of CspC, a CspA family protein, is critical for modulating the expression of the T3SS genes upon temperature fluctuation [20]. The low temperature-inducible expression of TypA, a suppressor of T3SS, may provide another explanation for the regulatory mechanism of T3SS mediated by temperature shifts. Additionally, we also found that *typA* was upregulated in *P. aeruginosa* in the biofilm lifestyle, which may contribute to the downregulated T3SS in the biofilm of *P. aeruginosa*. However, we also found that T3SS-inducing conditions, such as induction with 5 mM EGTA and direct contact with host cells, stimulate the expression of *typA*. Moreover, our earlier study indicates an increased expression of *typA* during the infection of mouse acute pneumonia [29]. This is not the first case where the repressor of T3SS was stimulated in response to the inducing signals of T3SS in *P. aeruginosa*. Elevated *ptrA* expression was also observed during the infection of mouse burn wounds [12]. ExsD was able to re-associate with and inhibit the function of ExsA at 37 °C, but not at 25 °C, whereas the T3SS was upregulated at 37 °C compared to 25 °C [35]. These observations support a hypothesis that some T3SS repressors, such as TypA, are induced under T3SS-inducing conditions, limiting excessive T3SS activation. However, the detailed signals and mechanisms for upregulating *typA* remain unclear and need further investigation. Together, these findings suggest that tight regulatory mechanisms have evolved to control the energy-intensive type III secretion apparatus in response to specific environments in *P. aeruginosa*.

TypA, also named BipA (BPI-inducible protein A), is a member of the EF-G family of translational GTPases and was first identified as a responsive protein to bactericidal/permeability-increasing protein (BPI), a cationic antimicrobial protein secreted by human neutrophils [13,14]. TypA undergoes tyrosine phosphorylation in EPEC (enteropathogenic *E. coli*) strains, while the phosphorylation occurs in a strain-specific manner, as neither *Salmonella enterica* nor *E. coli* K-12 produced a tyrosine phosphorylated BipA/TypA [14]. It has been shown that TypA enhances the accurate and efficient ribosome assembly under low-temperature conditions in *E. coli* [31,36]. TypA is involved in modulating virulence determinants and stress responses in a range of pathogens. In *Yersinia pestis*, BipA enhances bacterial survival in vivo and promotes pathogenesis in a murine model of pneumonic plague infection [15]. In *S. enterica* serovar Typhi, TypA contributes to uptake and intracellular survival when in contact with human macrophages [16]. In *E. coli*, BipA is involved in regulating the production of extracellular K5 polysaccharide [17], flagella-mediated bacterial motility [14,18], the *espC* pathogenicity island and the T3SS pathogenicity island [18], as well as the interaction between EPEC and epithelial cells [14]. In the present study, we demonstrated that TypA functions as a negative regulator of T3SS in *P. aeruginosa*. However, the role of tyrosine phosphorylation in TypA function remains unclear and awaits further exploration. Further investigation is also needed to distinguish whether TypA-mediated T3SS repression depends on its ribosome-associated GTPase activity or merely the direct physical interaction with ExsA.

Of note, it has been reported that TypA modulates the pathogenesis in *P. aeruginosa* [26]. Using a *P. aeruginosa* strain PA14 background, Anke Neidig et al. found that T3SS-related genes were downregulated in the *typA* mutant [26]. However, in the present study, expression of T3SS and the cytotoxicity mediated by T3SS were up-regulated when *typA* was absent in *P. aeruginosa* strains, including PAK, PAO1 and PA14 strains. Such a differential effect of TypA on T3SS might be due to difference in the experimental conditions used in these two studies. In the previous study by Anke Neidig et al., the transcriptional levels of T3SS were determined during the *P. aeruginosa* interaction with the amoeba *Dictyostelium discoideum*. In our study, the expressions of T3SS were examined under traditional T3SS-inducing conditions with 5 mM EGTA. Of note, differences in the strain background could not be ruled out. Even though PA14 was also used in our study, in which *typA* also exhibits a repressive function on T3SS, differences may also exist in PA14 during their transmission in different labs. The detailed molecular mechanisms related to the distinct functions of TypA in different *P. aeruginosa* strains remain poorly understood and require additional investigation.

Our mass spectrometry analysis revealed 417 proteins with potential interactions with ExsA (Table S1). Interestingly, MgtE and RsmA, two known T3SS regulators, were detected among them. *mgtE* encodes a magnesium transporter and was found to modulate T3SS gene expression by inhibiting ExsA activity [37]. Another study further demonstrated that MgtE stimulates regulatory RNAs RsmY and RsmZ transcription via the GacAS two-component system, ultimately resulting in the inhibition of *exsA* translation and expression of T3SS genes [38]. RsmA, a CsrA-family RNA-binding protein in *P. aeruginosa*, acts as a global post-transcriptional modulator of bacterial gene expression [39]. Although RsmA exerts a positive effect on T3SS gene expression, the exact molecular mechanism of activation has not been characterized [39]. Our preliminary mass spectrometric analysis demonstrated an interaction between ExsA and MgtE or RsmA. Currently, we are making efforts to understand whether MgtE/RsmA-mediated regulation of T3SS also occurs via interaction with ExsA. Interestingly, ExsD, a well-known ExsA binding protein, was also detected in the mass spectrometric analysis (Table S1). However, based on these data, at present, we cannot speculate whether TypA inhibits the binding of ExsD to ExsA, which will be the subject of future studies. On the other hand, our EMSA assays demonstrate that TypA blocks ExsA-DNA interaction. Nevertheless, our current data cannot resolve whether TypA disrupts ExsA homodimerization or directly shields its DNA-binding domain. Further structural and biochemical experiments will be needed to distinguish these models in follow-up work.

## 5. Conclusions

In summary, this work identifies TypA as a previously uncharacterized binding protein of ExsA and elucidates its repressive role on T3SS by modulating the DNA-binding capacity of ExsA in *P. aeruginosa*. We also revealed that the transcriptional level of *typA* was induced by low temperature, low calcium, contact with host cells, and biofilm growth status in *P. aeruginosa*. The importance of this study lies in the identification of a novel T3SS negative regulator and the elucidation of its regulatory circuitry. Our work advances the understanding of virulence control in *P. aeruginosa*.

## Data Availability

The original contributions presented in this study are included in the article/Supplementary Materials. Further inquiries can be directed to the corresponding author.

## Supplementary Materials

The following supporting information can be downloaded at https://www.mdpi.com/article/10.3390/microorganisms14091942/s1, Figure S1: Coomassie brilliant blue staining of purified TypA-Flag-His proteins. Recombinant TypA-Flag-His protein was separated by SDS-PAGE gel and stained with Coomassie brilliant blue. Lanes 1–4, samples were eluted successively with 1 mL elution buffer; M, marker; Figure S2: *typA* represses T3SS in PA14 and PAO1 strains. (A,B) Expression of ExoSand ExoUor secretion of ExoSin the indicated strains. (C,D) Relative mRNA levels of the indicated genes in the indicated strains. Total RNA was purified from bacteria at an OD_600_ of 1.0, and mRNA levels were determined by real-time qPCR using the ribosomal encoding gene *rpsL* as an internal control. (E,F) Cytotoxicity of indicated strains by crystal violet assay. ns, not significant, *, *p* < 0.05; **, *p* < 0.01; by Student’s *t*-test.; Figure S3: Relative mRNA levels of the indicated genes in PAK cultured at 37 and 20 °C. Total RNA was purified from bacteria at an OD_600_ of 1.0 cultured at 37 or 20 °C, and mRNA levels were determined by real-time qPCR using the ribosomal encoding gene *rpsL* as an internal control. *, *p* < 0.05 by Student’s *t*-test; Table S1: The potential proteins interacting with ExsA with mass spectrometry analysis; Table S2: Bacterial strains and plasmids used in this study [19,24,40,41,42,43,44]; Table S3: Primers used in this study.
