# Supplementary material for: TypA Interacts with ExsA to Suppress Type III Secretion System in Pseudomonas aeruginosa
Source: Microorganisms. 2026 Sep 2;14(9):1942. doi: 10.3390/microorganisms14091942 (PMC13609732; doi:10.3390/microorganisms14091942)
Supplement: Supplementary file 1 [file microorganisms-14-01942-s001.zip › supplementary materials.pdf]

Figure S1

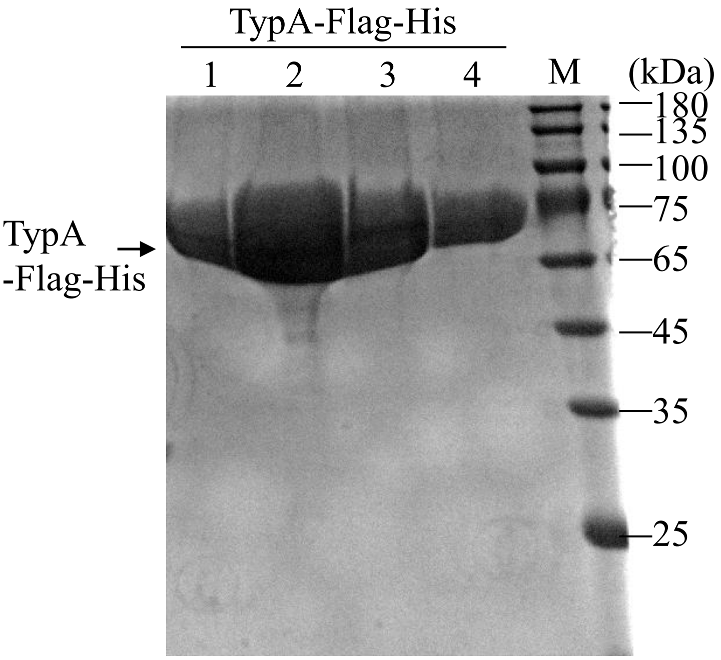

Figure S1. Coomassie brilliant blue staining of purified TypA-Flag-His proteins. Recombinant TypA-Flag-His protein was separated by SDS-PAGE gel and stained with Coomassie brilliant blue. Lanes 1-4, samples were eluted successively with 1 mL elution buffer; M, marker.

Figure S2

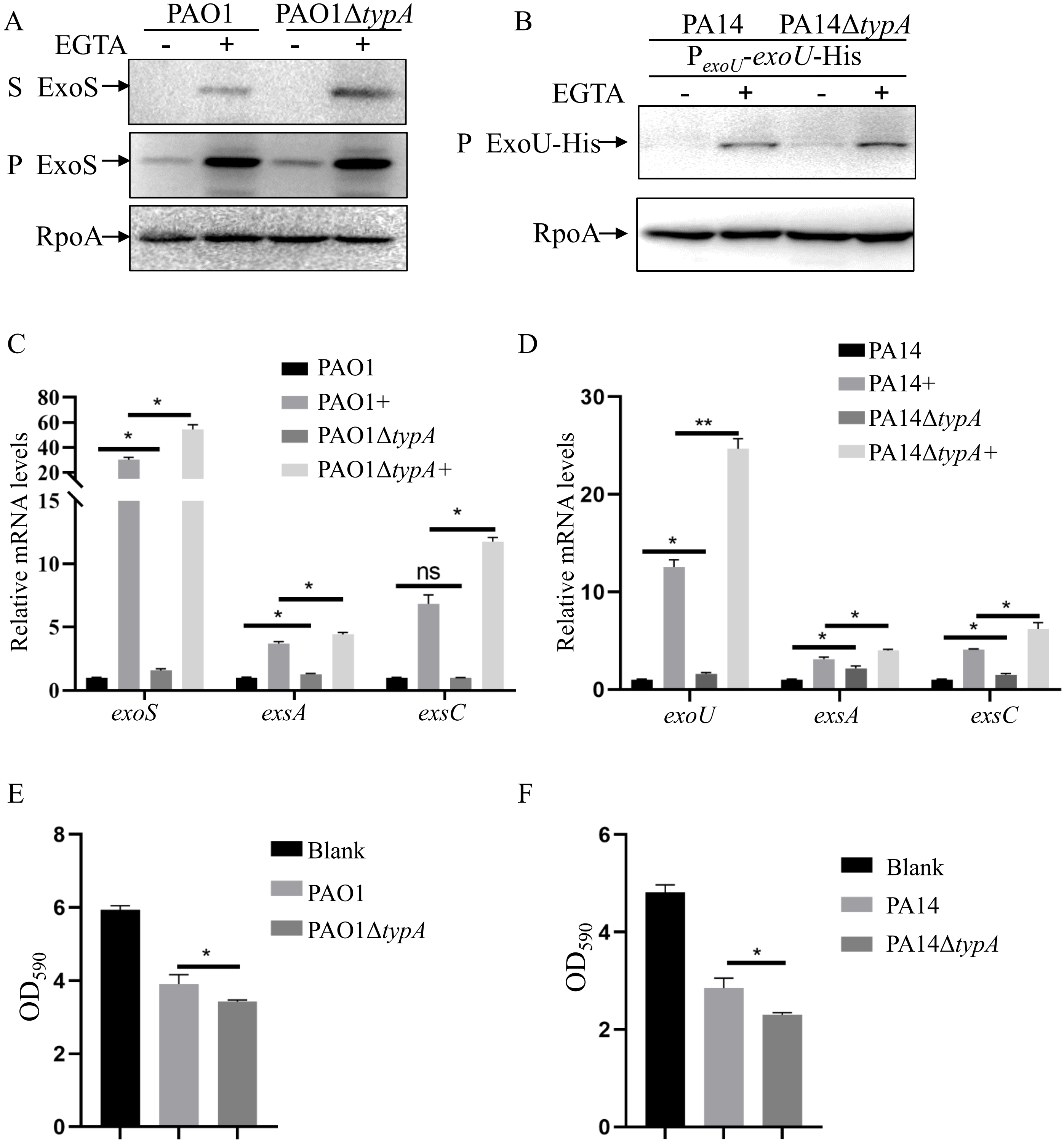

Figure S2. *typA* represses T3SS in PA14 and PAO1 strains. (A,B) Expression of ExoS and ExoU or secretion of ExoS in the indicated strains. **(C,D)** Relative mRNA levels of the indicated genes in the indicated strains. Total RNA was purified from bacteria at an OD_{600} of 1.0, and mRNA levels were determined by real-time qPCR using the ribosomal encoding gene *rpsL* as an internal control. **(E,F)** Cytotoxicity of indicated strains by crystal violet assay. ns, not significant, *, $p<0.05$; **, $p<0.01$; by Student's *t*-test.

Figure S3

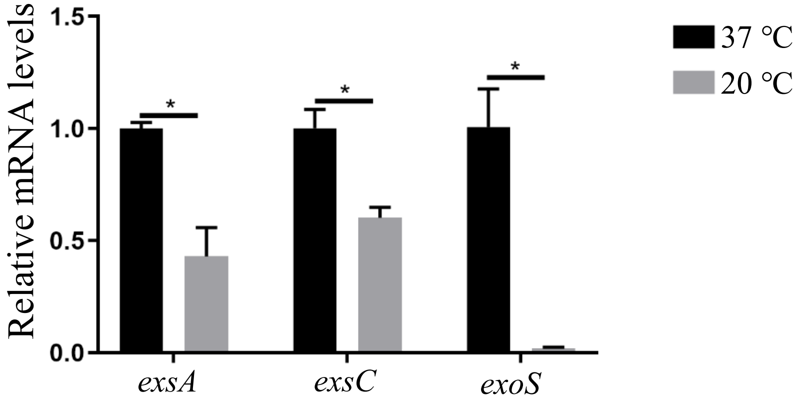

Figure S3. Relative mRNA levels of the indicated genes in PAK cultured at 37 and 20 ℃. Total RNA was purified from bacteria at an OD_{600} of 1.0 cultured at 37 or 20 ℃, and mRNA levels were determined by real-time qPCR using the ribosomal encoding gene *rpsL* as an internal control. *, $p<0.05$ by Student's *t*-test.

Table S2. Bacterial strains and plasmids used in this study [19,24,40–44].

Strains or plasmids	Description	Source or reference
**strains**		
DH5α	F$^-$ ϕ 80d*lacZ*ΔM15 *endA1 recA1 hsdR17*(r_K^- m_K^+) *supE44 thi-1 relA1* Δ(*lacZYA-argF*)*U169 gyrA96 deoR*	TransGen
S17-1	RP4-2 Tc::Mu Km::Tn*7* Tp^r Sm^r Pro Res$^-$ Mod$^+$	Stratagene
BL21 (DE3)	F$^-$ *ompT hsdSB (rB-, mB-) gal dcm* (DE3)	invitrogen
RS	Δ(*mcrA)*183 Δ(*mcrCB-hsdSMR-mrr*)173 *endA1 supE44 thi-1 recA1 gyrA96 relA1 lac* [F′ *laqI*^{q} *bla lacZ* Kan^r]	Stratagene
PAK	Wild type *P. aeruginosa* strain	David Bradley
PAKΔ*exsA*	PAK with *exsA* gene deleted	[40]
PAKΔ*typA*	PAK with *typA* gene deleted	This study
PA14	Wild type *P. aeruginosa* strain	Liberati, N.T.,2006
PA14Δ*typA*	PA14 with *typA* gene deleted	This study
PAO1	Wild type *P. aeruginosa* strain	[41]
PAO1Δ*typA*	PAO1 with *typA* gene deleted	This study
**Plasmids**		
pUCP21	Shuttle vector between *E. coli* and *P. aeruginosa*; Amp^r	[42]
pUCP21-*exsA*-Flag-His	Flag-His-tagged *exsA* gene from PAK in pUCP21; Amp^r	This study
pUCP24	Shuttle vector between *E. coli* and *P. aeruginosa*; Gm^r	[42]
pUCP24-*typA*-Flag	Flag-tagged *typA* gene from PAK in pUCP24; Gm^r	This study
pUC18T-mini-Tn7T-*typA*-Flag	Flag-tagged *typA* gene from PAK in pUC18T-mini-Tn7T; Gm^r	This study
pUCP19-GFP-His	His-tagged *gfp* gene in pUCP19; Amp^r	This study
pMMB67EH	Shuttle vector between *E. coli* and *P. aeruginosa*; Amp^r	[43]
pMMB67EH-*exsA*-His	His-tagged *exsA* gene from PAK in pMMB67EH; Amp^r	This study
pMMB67EH-GST-*exsA*-His	GST-His-tagged *exsA* gene from PAK in pMMB67EH; Amp^r	This study
pEX18Tc	Gene knockout vector; Tc^r	[19]
pEX18Tc-*typA*	*typA* gene deletion on pEX18Tc; Tc^r	This study
pDN19-P_{exsC}-*lacZ*	*exsC* promoter fused to promoterless *lac*Z on pDN19*lac*ZΩ; Sp^r, Sm^r, Tc^r	[44]

pET41a	Expression vector, Kan^r	Novagen
pET41a-GST-His	GST gene cloned into pET41a expression vector, Kan^r	This study
pET28a	expression vector, Kan^r	Novagen
pET28a-*exsA*-His	*exsA* gene of PAK cloned into pET28a expression vector, Kan^r	[24]
pET28a-GST-*exsA*-His	*exsA* gene of PAK cloned into pET28a expression vector, Kan^r	This study
pET28a-*typA*-Flag-His	Flag-tagged *typA* gene of PAK cloned into pET28a expression vector, Kan^r	This study
pBT	Vector for bacteria two-hybrid, $Ch1^r$	Stratagene
pBT-*exsA*	*exsA* from PAK cloned into pBT, $Ch1^r$	This study
pTRG	Vector for bacteria two-hybrid, Tc^r	Stratagene
pTRG-*typA*	*typA* from PAK cloned into pTRG, Tc^r	This study
pTRG-*exsD*	*exsD* from PAK cloned into pTRG, Tc^r	This study

Table S3. Primers used in this study.

Primer[a]	Sequence 5'-3'[b]	Use
typA-UF	CGGAATTCGCCCACTACCTGTGGGAGAA	for *typA* deletion
typA-UR	CGGGATCCAAGTCTTGTCTCGTTTAACG	
typA-DF	CGGGATCCACCGTTGCCGTACCGGCGCG	
typA-DR	CCAAGCTTCGCGGCTCACCAGCAT	
exsA-Flag-His-F	GCGAGCTCGTACGACGGGAAGTG	pUCP21-*exsA*-Flag-His
exsA-Flag-His-R	CGGAATTCTCAATGGTGATGGTGATGATGCTTGTCATCGTCGTCCTTGTAATCGTTATTTTAGCCCGGCATT	
exsA-His-F	CGGAATTCATGCAAGGAGCCAAATC	pMMB67EH-*exsA*-His
exsA-His-R	CCAAGCTTTCAATGGTGATGGTGATGATGGTTATTTTAGCCCG	
typA-Flag-His-F	TTAAGAAGGAGATATACCATGGTGATCGAAAATCTGCGCAACA	pET28a-*typA*-Flag-His
typA-Flag-His-R	GGTGGTGGTGGTGGTGCTCGAGCTTGTCATCGTCGTCCTTGTAATC	
typA-Flag-F	CGGAATTCGGTGATCGAAAATCTGC	pUCP24-*typA*-Flag
typA-Flag-R	CCAAGCTTTTACTTGTCATCGTCGTCCTTGTAATCGACCTTGCTGCGCTC	
mini-P_{typA}-*typA*-F	CGGGATCCACGAACTCGGCCAGGAC	pUC18T-mini-Tn7T-*typA*-Flag
typA-Flag-R	CCAAGCTTTTACTTGTCATCGTCGTCCTTGTAATCGACCTTGCTGCGCTC	
pBT-*exsA*-F	CGGAATTCCATGCAAGGAGCCAAATC	pBT-*exsA*
pBT-*exsA*-R	CGGGATCCTCAGTTATTTTTAGCC	
pTRG-*typA*-F	CGGGATCCGTGATCGAAAATCTGC	pTRG-*typA*
pTRG-*typA*-R	CGGAATTCTTAGACCTTGCTGCGCTC	
pTRG-*exsD*-F	CGGGATCCATGGAGCAGGAAGACGA	pTRG-*exsD*
pTRG-*exsD*-R	CGGAATTCTCAGCTCTGCCAGTAGAA	
GST-*exsA*-F	CGGGATCCCAAGGAGCCAAATCTCTT	pMMB67EH-GST-*exsA*-His
GST-*exsA*-R	CCCAAGCTTTCAATGGTGATGGTGATGATGGTTATTTTTAGCCCGGC	
GST-A-F	TTAAGAAGGAGATATACCATGGTGTCCCCTATACTAGGTTATTGGA	pET28a-GST-*exsA*-His
GST-A-R	GGTGGTGGTGGTGGTGCTCGAGGTTATTTTTAGCCCGGCATTC	
P_{exsC}-F	CGCCTCCTAAAGCTCAGCGCATGC	for EMSA
P_{exsC}-R	ATGAAGGACGTCCTGCAGCTCATCC	
algD-F	CAGGGGTGTCGGAGGGACGAACGGTA	for EMSA
algD-R	ACGGCTATTACTTCAGCGCCGAGCAATC	
qPCR primer		
q-*exoS*-F	GCATATTCAATCGCTTCAG	RT-qPCR of *exoS*
q-*exoS*-R	CCTCAATCTGTCCCAAAC	
q-*exsA*-F	GCTATGTCGTAAGTACCA	RT-qPCR of *exsA*
q-*exsA*-R	GAAGCCTTGTAGAAACTG	
q-*exsC*-F	ATGGATTTAACGAGCAAGGTCAA	RT-qPCR of *exsC*

q-*exsC*-R	GAGGGACAGGGAAGGCAAA	
q-*rpsL*-F	CAAAACTGCCCGCAACGT	RT-qPCR of *rpsL*
q-*rpsL*-R	TTTCGGCGTGGTGGTGTAT	
q-*typA*-F	CGACTTCCCGATCGTCTACG	RT-qPCR of *typA*
q-*typA*-R	GTGGTGGCCCATGATCTTCA	
q-*capB*-F	ATGTCGAATCGTCAGAAC	RT-qPCR of *capB*
q-*capB*-R	GATGGAACGGAAGTGAAC	

a: F: forward; R, reverse; U, upstream of specific gene; D, downstream of specific gene; b: The underlines are the sites of restriction enzymes.
